# Animal Agrocrime: An Overlooked Biological Threat

**DOI:** 10.1089/hs.2022.0144

**Published:** 2023-09-26

**Authors:** Daniel Donachie, Fanny Ewann, Frédéric Poudevigne

**Affiliations:** Daniel Donachie, BVMS, MRCVS, is Programme Manager – Emergency Management, Preparedness and Resilience Department, World Organisation for Animal Health, Paris, France.; Fanny Ewann, PhD, is a Specialized Officer, Bioterrorism Prevention Unit, CBRNE & Vulnerable Target Sub-Directorate, International Criminal Police Organization (INTERPOL), Lyon, France.; Frédéric Poudevigne, DMV, MSc, is an Emergency Management Specialist, Emergency Management Center, Food and Agriculture Organization of the United Nations, Rome, Italy.

**Keywords:** Agrocrime, Agroterrorism, Law enforcement, Veterinary services, Animal health

## Background

The COVID-19 pandemic, suspected to have originated from spillover events,^1^ has significantly increased the visibility of biological threats, whether their origins are natural, accidental, or deliberate.^2^ The pandemic has also revealed vulnerabilities and gaps in emergency preparedness planning that were exploited by criminals during the crisis. However, how different would the pandemic have looked if it had been deliberately caused? In April 2020, the United Nations Secretary-General António Guterres warned that “the weaknesses and lack of preparedness exposed by this pandemic provide a window onto how a bioterrorist attack might unfold.”^3^ Imagine the chaos that would have unfolded—how would we have recognized the signs of a deliberate biological event affecting animals and humans, and how would we have taken a One Health approach^4^ to such an event?

The COVID-19 pandemic response was led by the public health community, but we also saw strong contributions from veterinary and law enforcement professionals. Veterinary professionals contributed to public health epidemiological investigations, laboratory testing of human specimens for the virus, and experimental infection of animals to further scientific evidence surrounding the virus, while simultaneously trying to maintain their critical functions in safeguarding animal health, welfare, and veterinary public health.^5^ Law enforcement officials also played an important role in the pandemic by supporting efforts to control the disease and handle criminals who took advantage of the situation, for example, through fraudulent activity, cyberattacks, and counterfeit medical supplies and medicine.^6,7^ In many countries, law enforcement officials took on new or unfamiliar duties that exposed them to infected people, often with minimal guidance and preparedness.^8^

Public health, law enforcement, and veterinary professionals faced significant pressures during this natural disease outbreak. However, what would the expectations have been if they were asked to respond to a deliberate biological event targeting governments and the public through livestock? Crime and terrorism surrounding animal health are often overlooked threats but can have substantial impacts on animal health and welfare, public health, food security, food authenticity, and even national security.^9^ Animal diseases can affect the animal and animal product trade, and their ability to infect wildlife can make it difficult for them to be contained within a single region, as demonstrated by the recent African swine fever outbreaks spreading in several parts of the world.^10^

## Why Is the Animal Health Sector Vulnerable?

The animal health sector has several unique characteristics that render it vulnerable and attractive to crime and terrorism.^11^ Animal diseases, including those of a zoonotic nature, have the ability to spread quickly on a farm and at national, regional, and international levels. Agriculture also contains complex interwoven supply chains at both national and international levels. Animals can move from farms to markets and slaughterhouses, with their products subsequently moving on to various food processing businesses before being made available to consumers. Moreover, agriculture has evolved to increase production and efficiency through intensive farming practices. Animals can move across vast distances quickly and are often born, fattened, and slaughtered in different locations.^12^ Smaller farming units around the globe are generally more exposed to disease introduction because suitable prevention measures are lacking.^13^ Unlike critical infrastructure, farms have few or nonexistent security measures.^14^ We know the importance of animals for food security^15^ and livelihoods,^16^ and it is therefore vital that agriculture be treated as a national security priority.^17,18^ However, threats to animal health are often not on the radar of law enforcement agencies and subsequently considered a low priority.^9^

Throughout history, animal disease outbreaks have catalyzed civil and political unrest. For example, in 1896 and 1897, the now eradicated rinderpest virus swept through southern Africa, deeply affecting the transport system, food supplies, and the economy. Local leaders emerged who were willing to use grievances, widespread suspicion, and rumors stemming from rinderpest to mobilize the public toward revolt.^19^ In World War II, on both sides of the conflict, some countries developed offensive biological weapons programs, including those that targeted livestock and used zoonotic pathogens.^20^

The ubiquitous nature of high-consequence animal pathogens in nature and in laboratories, combined with their potential impacts, makes them attractive to criminals and terrorists for use as bioweapons. Most pathogens that have been used as bioweapons are animal pathogens.^21^ If a comparison is made between the Australia Group, which identifies exports that must be controlled to not contribute to the spread of chemical and biological weapons,^22^ and the list of notifiable diseases of the World Organisation for Animal Health (WOAH),^23^ there are many pathogens in common, such as foot-and-mouth disease, highly pathogenic avian influenza, and anthrax. In fact, 80% of the pathogens with biological weapons potential are zoonotic, which further demonstrates the risks to One Health if animal pathogens were used as weapons, particularly for public health.^21^

## Agrocrime and Agroterrorism

Regarding animals, agrocrime can be defined as an unlawful act or omission concerning animals or animal products that violates legislation and has negative consequences on animal health, animal welfare, public health, food safety, food authenticity, or national security.^24^ This can include various criminal activities such as:

- Falsification of veterinary and animal products
- Animal welfare crimes
- Illicit wildlife use
- Circumventing disease control measures through smuggling of animals and their associated products
- Deliberate release of animal pathogens

Although research is limited regarding the global risk of disease spread linked to this crime area, animal and zoonotic cases and outbreaks have been linked to agrocrime.^25-27^ Such criminal activities expose directly both perpetrators^28^ and law enforcement officials acting on the front lines to prevent and fight agrocrime activities.^29^ Because many of these crimes are seen as highly lucrative and entailing low penalties, they have the potential to be linked to organized crime^30^ or to the financing of terror groups^31^ directly or associated with other criminal activities, such as smuggling of weapons, money laundering, and human trafficking.^32^

While methods of operation can be similar, agrocrime is motivated by personal or financial gain, whereas agroterrorism, a subset of agrocrime, can be defined as the deliberate release of viruses, bacteria, toxins, or other harmful agents to cause illness or death in animals with the intent to intimidate or coerce governments or civilian populations to further political or social objectives.^9^

In the response to an agrocrime event, veterinary professionals are responsible for epidemiological investigation, protecting animal health and welfare, preventing further disease outbreaks and cases, and furthering the scientific base for future prevention. Law enforcement officials, on the other hand, conduct criminal investigations; maintain public order; prevent further acts of crime and terrorism; and identify perpetrators, accomplices, victims, and witnesses. While these activities appear distinct, they must be conducted in a coordinated manner to achieve the common goal of protecting public health and safety.^33^

Protecting the health and safety of the public is a goal also shared by public health authorities who must cooperate with law enforcement officials in preparedness and response efforts for health emergencies including zoonoses.^34^ In the event of a zoonotic agrocrime or agroterrorism incident, it is essential to have a strong, well-coordinated One Health approach that includes the security sector.^35^

The COVID-19 pandemic has brought the public health and law enforcement sectors closer together, but challenges remain in integrating law enforcement into the One Health approach, particularly the cooperation between law enforcement and veterinary service professionals.^36^ Differences in terminologies, perspectives, expertise, and competing priorities make it harder for them to cooperate on questions related to the spread of diseases as part of animal-related crimes. Veterinary professionals may not necessarily see how crimes are associated with disease spread, and law enforcement officials may not understand how crimes could cause disease introduction or spread, especially if the disease does not directly affect human health. Another challenge is that formal collaborative frameworks are often absent, meaning that there are no agreements, protocols, or guidance in place describing how to work together. These frameworks are essential for sharing intelligence needed to prevent and respond to agrocrime.^9^

The 2 sectors can, however, take steps to overcome these challenges. First, they can begin with a dialogue to build relationships and understand their respective roles and responsibilities. It is also crucial to identify common priority areas to prevent and control agrocrime, which can then be formalized into a cooperation agreement between veterinary services and law enforcement to share information and intelligence based on agreed indicators and triggers. Such an agreement can be used to undertake joint activities including threat assessments, training workshops, and simulation exercises. This relationship, fostered in peacetime, can be operationalized in emergency response situations, whether for natural, accidental, or deliberate animal disease outbreaks.^9^

During an agrocrime incident, preestablished indicators must lead to coordinated action. Indicators for the veterinary sector can include disease outbreaks with unknown or unusual epidemiological patterns. For the law enforcement sector, this can be any suspicious activity around relevant facilities, security breaches, intelligence or police reports, and credible threats. It is essential that the 2 sectors proactively share information to inform their respective operations, and if necessary, instigate joint responses that could consist of joint credibility threat assessments, joint criminal and epidemiological investigations (including joint interviews), and common public messaging.^33^ The veterinary and law enforcement sectors could build upon what exists already between the public health and law enforcement sectors and explore similar mechanisms of cooperation.^34,37^

## International Partnerships to Prevent Agrocrime

Since October 2018, WOAH, the International Criminal Police Organization (INTERPOL), and the Food and Agriculture Organization of the United Nations (FAO) have been working on a joint project aimed at building resilience against agrocrime and agroterrorism by fostering coordination between law enforcement and veterinary services.^6,38^ This project assesses the global landscape for agrocrime and agroterrorism,^39,40^ identifies solutions to improve preparedness for these threats,^41^ and delivers capacity-building activities such as training workshops and simulation exercises.^42^

In March 2022, WOAH and INTERPOL signed a cooperation agreement aimed at working together within their respective mandates, in the areas of agrocrime, agroterrorism and incidents involving animal or zoonotic pathogens, and biological toxins affecting animals. Both organizations will share data and expertise to support their respective memberships and cooperate technically through capacity-building and training activities.^6,38^ They hope that this cooperation at the international level can transpire to cooperation at the national level for their respective memberships. To date, the 2 organizations have coorganized workshops on agrocrime and disinformation, facilitated the exchange of experts across the law enforcement and veterinary sectors, and are mapping future areas of cooperation such as wildlife crime, falsified veterinary products, disease intelligence, and interoperability in incident management systems.

WOAH, INTERPOL, and FAO are also looking toward the future by strengthening their cooperation with the public health sector through the World Health Organization (WHO) by monitoring global trends to identify and anticipate current and future shared threats, develop associated prevention and mitigation measures, and provide relevant guidance for their respective sectors. The 77th Session of the United Nations General Assembly also recommended, through a resolution on cooperation between INTERPOL and the United Nations, that both organizations should work closer together, particularly WHO and INTERPOL, to cooperate and exchange information about events on biological incidents whether from natural, accidental, or deliberate origins.^43^ The cooperation between WOAH, INTERPOL, and FAO can also be applied to the United Nations Environmental Programme, the newest member of the “quadripartite,” to further integrate security in the One Health approach.

Lastly, WOAH, INTERPOL, and FAO continue to support the work of the G7-led Global Partnership Against the Spread of Weapons and Materials of Mass Destruction, which works at the health security interface to deliver capacity-building programs and mitigate global biological threats and safeguard global health security.^44^

## Conclusion

Bioterrorism threats are often addressed from a public health perspective, with much less attention given to the direct or indirect threat to animals in preparing and planning for such events. A One Health approach must be taken that brings all relevant actors and sectors to the table. In an all-hazards approach to biological threats, the veterinary and law enforcement sectors have important roles to play alongside public health, and they must jointly plan to prevent and respond to criminal activities affecting primarily animal health in the form of agrocrime acts, including agroterrorism. Cooperation exists already between the public health and veterinary sectors and between law enforcement and public health, particularly through the COVID-19 pandemic, and lessons identified from this cooperation can be applied to build and strengthen partnerships between the veterinary and law enforcement sectors.

Beyond the contribution of livestock to countries' agricultural gross domestic product, for communities in low-medium income countries, livestock may serve multiple functions with high potential impacts that are not always captured by the gross domestic product (eg, food production, income generation, draft power, savings/insurance resource, manure for fertilizer, ceremonial value).^45^ Agriculture is critical to national security, and preparedness for agrocrime must also be integrated into contingency planning at both national and international levels. Preventing and tackling agrocrime and agroterrorism will contribute to mitigating the risk of spillover of diseases from animals into human populations.^14^ WOAH, FAO, and INTERPOL are taking concrete steps forward to support their members and the international community to be better prepared for agrocrime and global biological emergencies and disasters and cooperating with other One Health stakeholders.
